# A Review of Biomarkers in Delirium Superimposed on Dementia (DSD) and Their Clinical Application to Personalized Treatment and Management

**DOI:** 10.7759/cureus.38627

**Published:** 2023-05-06

**Authors:** Saira Jahangir, Manoj Allala, Armughan S Khan, Veronica E Muyolema Arce, Anandkumar Patel, Karsh Soni, Alireza Sharafshah

**Affiliations:** 1 Neurology, Dow University of Health Sciences, Civil Hospital Karachi, Karachi, PAK; 2 Internal Medicine, Mediciti Institute of Medical Sciences, Medchal, IND; 3 Internal Medicine, Midwest Sleep and Wellness, Gurnee, USA; 4 Internal Medicine, JC Medical Center, Florida, USA; 5 Medicine, Universidad de Guayaquil, Guayaquil, ECU; 6 Medicine, Maharshi Hospital Private Limited, Surendranagar, IND; 7 Neurology, Shalby Hospitals Naroda, Ahmedabad, IND; 8 Neurology, Grodno State Medical University, Ahmedabad, IND; 9 Genetics, Marie Curie Science Research Center, New York City, USA

**Keywords:** neurology, psychiatry, delirium, dementia, geriatrics, bioinformatics, delirium superimposed dementia, hsa-mir-17

## Abstract

Delirium superimposed on dementia (DSD) occurs when patients with pre-existing dementia develop delirium. This complication causes patients to become impaired, posing safety concerns for both hospital staff and patients. Furthermore, there is an increased risk of worsening functional disability and death. Despite medical advances, DSD provides both diagnostic and therapeutic challenges to providers. Identifying at-risk patients and providing personalized medicine and patient care can decrease disease burden in a time-efficient manner.

This review delves into bioinformatics-based studies of DSD in order to design and implement a personalized medicine-based approach. Our findings suggest alternative medical treatment methods based on gene-gene interactions, gene-microRNA (miRNA) interactions, gene-drug interactions, and pharmacogenetic variants involved in dementia and psychiatric disorders.

We identify 17 genes commonly associated with both dementia and delirium including apolipoprotein E (ApoE), brain-derived neurotrophic factor (BDNF), catechol-O-methyltransferase (COMT), butyrylcholinesterase (BChE), acetylcholinesterase (AChE), DNA methyltransferase 1 (DNMT1), prion protein (PrP), tumor necrosis factor (TNF), serine palmitoyltransferase long chain base subunit 1 (SPTLC1), microtubule-associated protein tau (MAPT), alpha-synuclein (αS), superoxide dismutase 1 (SOD1), amyloid beta precursor protein (APP), neurofilament light (NFL), neurofilament heavy, 5-hydroxytryptamine receptor 2A (HTR2A), and serpin family A member 3 (ERAP3). In addition, we identify six main genes that form an inner concentric model, as well as their associated miRNA. The FDA-approved medications that were found to be effective against the six main genes were identified. Furthermore, the PharmGKB database was used to identify variants of these six genes in order to suggest future treatment options.

We also looked at previous research and evidence on biomarkers that could be used to detect DSD. According to research, there are three types of biomarkers that can be used depending on the stage of delirium. The pathological mechanisms underlying delirium are also discussed. This review will identify treatment and diagnostic options for personalized DSD management.

## Introduction and background

Dementia is a progressive cognitive decline that significantly impairs an individual’s well-being and social function [1]. In contrast, delirium is characterized by an acute, transient, usually reversible, altered mental status that features inattention and an altered level of consciousness [2]. Each condition increases the risk of the other. Neuropsychiatric symptoms such as hallucinations, delusions, aggression, and sleep problems often persist as dementia progresses [3]. Delirium contributes to increased hospitalization, functional disability, and mortality, impacting healthcare utilization and costs. Despite medical and nursing interventions, 70% of individuals with delirium remain undiagnosed [4].

While delirium and dementia are distinct clinically, delirium superimposed on dementia (DSD) occurs when a person with pre-existing dementia develops delirium [5]. Delirium may cause hospitalized patients to become violent; therefore, identifying at-risk patients can enhance the safety of both patients and staff [6]. Three motor subtypes of delirium aid clinicians in diagnosis: 1) hyperactive delirium, characterized by restlessness, irritability, belligerence, or agitation; 2) hypoactive delirium, causing lethargy, reduced alertness, decreased motor activity, and unawareness; and 3) mixed delirium, exhibiting both hypoactive and hyperactive symptoms [2].

Quality in medicine is synonymous with patient safety. In our caring society, patients with dementia require high-quality, safe care. Traditionally, patient safety measures have focused on younger patients, particularly in high-stakes fields such as surgery and obstetrics, where concerns may be more apparent. However, it is essential to concentrate on vulnerable elderly individuals with dementia. Enhancing safety for dementia patients benefits all patients, primarily by promoting person-centered care and improving the hospital environment. Acute care systems for complex patients with multiple comorbidities warrant increased attention to address this issue [7].

Healthcare-related harm often manifests as geriatric syndromes, including falls, delirium, incontinence, and functional decline in elderly individuals [8-9]. Raising awareness and understanding of these issues is crucial. A “low expectation” mindset, which conflicts with the necessary “safety culture,” makes the presentation and identification of mild adverse events in dementia patients even more challenging [10]. Preventing delirium in an elderly dementia patient should be as critical as preventing wound infection in a young surgical patient [11-12].

Due to the inter-related nature of dementia and delirium, diagnosing and prognosing individuals based on personalized medicine can be time-efficient, cost-effective, precise, and molecularly targeted. Such studies may pave the way for DSD treatment without resorting to physical or violent interventions. This review focused on bioinformatics-based investigations of DSD and comprehensively examined the literature. The aim was to introduce novel gene-gene interactions (GGI), protein-protein interactions, gene-drug interactions (GDI), and gene-microRNA (miRNA) interactions (GMI). Discoveries in GMI may help identify circulating agents, such as miRNAs and long noncoding RNAs, in the plasma of individuals suspected of having DSD. GDIs can introduce potential candidate drugs to reduce aggressive actions and alleviate negative manifestations associated with DSD.

## Review

Bioinformatics for delirium 

Some 17 genes are common between dementia and delirium, including apolipoprotein E (ApoE), brain-derived neurotrophic factor (BDNF), catechol-O-methyltransferase (COMT), butyrylcholinesterase (BChE), acetylcholinesterase (AChE), DNA methyltransferase 1 (DNMT1), prion protein (PrP), tumor necrosis factor (TNF), serine palmitoyltransferase long chain base subunit 1 (SPTLC1), microtubule-associated protein tau (MAPT), alpha-synuclein (αS), superoxide dismutase 1 (SOD1), amyloid beta precursor protein (APP), neurofilament light (NFL), neurofilament heavy, 5-hydroxytryptamine receptor 2A (HTR2A), and serpin family A member 3 (ERAP3). The evidence revealed that six main genes are in the inner concentric model, including SNCA, APP, BDNF, DNMT1, APOE, and TNF. Furthermore, the miRNA associated with these genes was hsa-miR-17-5p, the most interactive and important miRNA. The fourth level of analyses investigated the GDIs for the six aforementioned genes in the concentric model. Drugs with unknown pharmacological actions were discarded, and considering this cutoff, TNF, APP, and DNMT1 genes exhibited reliable FDA-approved drugs (Table 1) [13-22].

**Table 1:** Gene-drug interactions of candidate genes for DSD. DSD, dementia superimposed delirium; APP, amyloid precursor protein; DB, DrugBank; DNMT1, DNA methyltransferase 1; TNF; tumor necrosis factor

Gene	Drug	Drug category	DB ID
TNF	Etanercept	Inhibitor	DB00005
TNF	Adalimumab	Inhibitor	DB00051
TNF	Infliximab	Inhibitor	DB00065
TNF	Certolizumab pegol	Neutralizer	DB08904
TNF	Pomalidomide	Inhibitor	DB08910
TNF	Golimumab	Antibody	DB06674
TNF	Foreskin keratinocyte [neonatal]	Agonist	DB10772
TNF	Glycyrrhizic acid	Antagonist	DB13751
APP	Florbetaben [18F]	Binder	DB09148
APP	Flutemetamol [18F]	Binder	DB09151
APP	Florbetapir [18F]	Binder	DB09149
APP	Gantenerumab	Binder/antagonist	DB12034
APP	Lecanemab	Binder	DB14580
DNMT1	Decitabine	Inhibitor	DB01262
DNMT1	Azacitidine	Inhibitor	DB00928

Based on the findings of the present review, personalized medicine for DSD was performed. The strategy to identify significant and actionable suggestions involved finding variants related to dementia, delirium, and other psychiatric manifestations. To search for plausible variants for future treatments of individuals with DSD based on personalized medicine, the PharmGKB database was used to determine variant annotations of the six candidate genes. The data revealed 205 variant annotations for BDNF, APOE, and TNF genes, while the other three had no variant annotations yet. After filtering for significant variants and applying specific cutoffs related to psychiatric phenotypes, 105 variant annotations were found. Among these, 15 annotations of BDNF and APOE genes indicated a relationship with DSD. Finally, seven variants were notable for further studies: rs11030104, rs6265, rs7103411, rs7124442, rs962369, rs405509, and rs429358 (Table 2) [13-22].

**Table 2:** Candidate variant based on pharmacogenetics perspective of DSD personalized medicine. APOE, apolipoprotein E; BDNF, brain-derived neurotrophic factor; DSD, delirium superimposed on dementia; HMG-CoA, hydroxymethylglutaryl-coenzyme A; PMID, PubMed Identification; PMCID, PubMed Central Identification; SSRI, selective serotonin reuptake inhibitors

Gene	Variant	Drugs	Association	p-Value	Literature
BDNF	rs11030104	Antipsychotics	Genotypes AG + GG is associated with increased resistance to antipsychotics in people with schizophrenia as compared to genotype AA.	0.00004	Zhang, et al., 2013 [13]
BDNF	rs6265	Analgesics; anti-inflammatory agents, non-steroids; ergot alkaloids; opioids; sumatriptan	Allele T is associated with decreased likelihood of headache disorders and substance withdrawal syndrome in people not taking analgesics, anti-inflammatory agents, non-steroids, ergot alkaloids, opioids and sumatriptan as compared to allele C.	0.001	Cargnin et al., 2014 [14]
BDNF	rs6265	Risperidone	Genotype TT is associated with decreased severity of weight gain due to risperidone in people with schizophrenia as compared to genotype CC.	0.02	Lane et al., 2006 [15]
BDNF	rs6265	Citalopram	Genotype CC is associated with decreased response to citalopram in people with depressive disorder, major as compared to genotype TT.	0.001	Domschke et al., 2010 [16]
BDNF	rs6265	Paroxetine	Genotypes CC + CT is associated with decreased response to paroxetine in women with depressive disorder, major as compared to genotype TT.	0.019	Wang et al., 2014 [17]
BDNF	rs6265	Methylphenidate	Genotype CC is associated with increased response to methylphenidate in children with attention deficit disorder with hyperactivity as compared to genotypes CT + TT.	0.0002	Kim et al., 2011 [18]
BDNF	rs6265	Antipsychotics	Genotypes CT + TT is associated with increased resistance to antipsychotics in people with schizophrenia as compared to genotype CC.	0.0013	Zhang et al., 2013 [13]
BDNF	rs7103411	Citalopram	Genotype TT is associated with decreased response to citalopram in people with depressive disorder, major as compared to genotype CC.	0.003	Domschke et al., 2010 [16]
BDNF	rs7124442	Citalopram	Genotype tt is associated with decreased response to citalopram in people with depressive disorder, major as compared to genotypes CC + CT.	0.01	Domschke et al., 2010 [16]
BDNF	rs962369	Escitalopram; nortriptyline	Allele C is associated with increased likelihood of suicidal ideation when treated with escitalopram or nortriptyline in people with depressive disorder, major as compared to allele T.	< 0.05	Perroud et al., 2009 [19]
APOE	rs405509	SSRIs	Genotype TT is associated with increased response to Selective serotonin reuptake inhibitors in people with depressive disorder, major as compared to genotypes GG + GT.	0.04	Yuan et al., 2020 [20]
APOE	rs429358	HMG-CoA reductase inhibitors	Genotype CC is associated with increased response to HMG-CoA reductase inhibitors in people with Alzheimer disease as compared to genotypes CT + TT.	< 0.01	Geifman et al., 2017 [21]
APOE	rs429358	Donepezil; galantamine; rivastigmine	Genotypes CC + CT is associated with decreased response to donepezil, galantamine and rivastigmine in people with Alzheimer disease as compared to genotype TT.	0.023	Braga et al., 2015 [22]

Pathophysiology of delirium

The exact pathophysiology of delirium is unknown, but it is thought to be a multifactorial process resulting in brain dysfunction. The attentional deficit that serves as a marker of delirium has diffuse localization within the brain. As evidenced by the discovery of symmetrical activity slowing on an electroencephalogram (EEG), which is a non-specific finding, delirium typically occurs from diffuse abnormalities in cortical and subcortical regions of the brain [23]. Inflammation, hypoxia, and oxidative stress increase the brain's exposure to toxins and create a state of low acetylcholine and high dopamine [24]. For example, in cases such as sepsis, where there is a systemic inflammatory response, proinflammatory cytokines released into the bloodstream can enter the central nervous system, leading to changes in the function of endothelial cells, decreased perfusion, activation of microglia, and death of neurons as well as neurotoxicity [25]. Transient episodes of subclinical hypoxia can result in reduced acetylcholine production, the primary neurotransmitter responsible for the functioning of the reticular activating system. The reticular activating system is vital in regulating alertness and attention, and its impairment is a crucial characteristic of delirium [24]. For example, the binding of anticholinergic medications to nicotinic and muscarinic receptors in the brain can worsen or trigger delirium by affecting cognitive function and arousal, providing further evidence for the role of cholinergic deficiency in delirium [25]. Furthermore, stress of any kind may suppress parasympathetic tone and enhance sympathetic tone, affecting cholinergic function and causing delirium. It mainly concerns older individuals, who are more vulnerable to reduced cholinergic transmission and are, therefore, at higher risk of developing delirium [26]. There is a suggestion that dopamine is a contributing part of the perceptual problems in delirium. In addition, oxidative damage may trigger the body to release dopamine, which can have this effect [24]. Irrespective of the root cause, delirium leads to a decline in the functioning of the cerebral hemispheres and the arousal mechanisms in the thalamus and reticular activating system located in the brain stem [26].

Epidemiology of delirium

Delirium is more prevalent among hospitalized elderly patients, with its occurrence depending on risk factors, clinical context, and diagnostic techniques. In conventional hospitals, delirium occurs in 14%-24% of patients before hospital admission and 6%-56% during hospitalization. Although delirium affects 1%-2% of the general population, this percentage increases to 14% among individuals over 85 years old [27-29].

Screening and diagnosis of delirium

Available delirium screening tests often exhibit low sensitivity and specificity in detecting dementia. The confusion assessment technique (CAM), based on the Diagnostic and Statistical Manual of Mental Disorders (DSM)-III-R criteria, demonstrates a 96% specificity and a 77% sensitivity in identifying delirium superimposed on dementia [30-36]. The CAM is widely used in both clinical and research settings and assesses patients through interviews and cognitive tests based on four criteria: 1) acute onset and fluctuating course; 2) inattention; 3) disorganized thinking; and 4) level of consciousness [7].

Delirium and dementia

Delirium is more commonly associated with psychosis, anxiety, and psychoactive medications that induce postural hypotension, drowsiness, and extrapyramidal symptoms [37]. While restraints are known to be dangerous, data suggest that older, cognitively impaired individuals, such as those with dementia, experience negative emotional responses like anger, anxiety, resistance, shame, and demoralization when restrained in a hospital setting. These individuals often recall the episode after discharge [7]. Physical restraints contribute to sleep loss and disrupt the sleep-wake cycle by keeping patients in one position. This disruption can alter melatonin secretion, a peptide hormone regulating circadian rhythm, potentially leading to delirium [38-39]. Several principles for managing patients with delirium include reorientation, cognitive stimulation, adequate nutrition and hydration, sensory stimulation, early mobilization, multidisciplinary medication review, restraint avoidance, visual and hearing screening, and optimizing pain management [40].

Prevention and management of delirium

Effective delirium management involves identifying and addressing predisposing risk factors, creating an optimal environment for brain recovery, minimizing neurological consequences, addressing patient concerns, monitoring for resolution, and educating sitters and caregivers about the risk of relapse [40-41]. Recent clinical recommendations advocate for multifaceted measures to prevent and treat delirium [40]. Restraints have been associated with psychoactive and sedative drug use, delirium, and language impairments. Delirious patients experience physical restraints more frequently than non-delirious patients; thus, reducing delirium may decrease restraint utilization. The ABCDE approach (Awakening and Breathing coordination, Delirium monitoring, and Exercise/early mobility) can help reduce delirium incidence [42]. When non-pharmacological treatments prove ineffective, antipsychotics can treat delirium [43]. Low-dose antipsychotics may alleviate the distressing symptoms of delirium or chemically restrain individuals at risk of causing harm to themselves or others [44-45]. The primary strategies for reducing complications in patients with DSD include pressure area care, fall prevention, early mobilization, judicious use of antipsychotics, prompt communication, and caregiver education [46].

GGIs, GMIs, GDIs, and personalized medicine

The current study assessed molecular investigations of DSD using bioinformatics analyses and obtained intriguing results. Here, we review publications that have reported evidence and suggestions based on various biomarkers for detecting DSD. According to Marcantonio et al., serum biomarkers indicating delirium can be divided into three primary groups: 1) those present or increased before disease initiation indicators, 2) those increasing with initiation and diminishing with recovery-disease markers, and 3) those arising in relation to disease consequences-and outcomes [47]. They also summarized previously reported biomarkers, including serum chemistries related to renal function, the APOE4 allele, the A9 allele of the dopamine transporter, serum anticholinergic activity, serum amino acids like tryptophan, serum melatonin, cytokines or inflammatory markers, hypercortisolism, neuron-specific enolase, and S-100 beta (tau protein) [47]. De Jonghe et al. found that melatonin administration reduces sundowning/agitated behavior in dementia patients [48]. This finding suggests that melatonin medication may share a common cause, such as disruption of the circadian rhythm, and have similar beneficial effects in delirium patients. In a comprehensive study, van den Boogard et al. evaluated 100 intensive care unit patients with or without delirium and discovered that the underlying mechanism regulating delirium onset differs between patients with or without infection/systemic inflammatory response syndrome [49].

Studies have also investigated the role of biomarkers in diagnosing and understanding the etiology of specific syndromes, such as Wernicke-Korsakoff syndrome [50] and Alzheimer’s disease [51-53]. Hov et al. found that cerebrospinal fluid (CSF) S100B levels were higher in patients with acute delirium and pathological Alzheimer’s disease biomarker P-tau levels, suggesting susceptibility induced by astrocytic activation and tau pathology [52]. Henjum et al. supported the concept of primed microglia in neurodegenerative diseases and proposed that a CSF neuroinflammation biomarker panel could be useful in preventing delirium by identifying patients at risk [53]. Genome-wide association studies have also discovered new loci for delirium risk, with McCoy Jr. et al. identifying linked variations on chromosomes 2, 6, 7, 10, and 20, associated with genes such as IL1, IRL1, IL18R1, IL18RAP, MIR4772, SLC9A2, SLC9A4, PDE7B, CDH26, FAM217B, PPP1R3D, and SYCP2 [54]. Vasunilashorn et al. found that APOE 4 genotype carrier status may alter the link between postoperative day two C-reactive protein levels and postoperative delirium (POD) [55]. Other studies have also investigated the associations between plasma markers of inflammation, coagulation, brain injury, and delirium duration in elderly hospitalized patients [56] and the interaction between APOE ε4 allele and vitamin D variations [57].

Research by Wang et al. analyzed delirium literature, revealing that few studies have shown a link between amyloid and tau biomarkers [58]. However, their separate study suggested that dysregulated innate immunity could deleteriously impact the blood-brain interface, causing delirium and exacerbating Alzheimer’s disease neuropathology [59]. Furthermore, Fong et al. found a relationship between CSF biomarkers for Alzheimer’s disease and POD in older adults, supported by the gradient effect, which indicates that when delirium occurs in individuals with any amyloid, tau, and neurodegeneration (ATN) biomarker, the severity of the delirium increases beyond the level of either delirium or ATN biomarkers alone [60]. In a recent study, Wang et al. conducted a meta-analysis to identify biomarkers for Alzheimer’s and related dementias associated with POD [61]. This meta-analysis revealed a connection between POD and specific inflammatory and neural damage biomarkers.

The bioinformatics findings of the current review suggested that BDNF and APOE variants might be candidates for personalized medicine-based detection of DSD. There are conflicting reports regarding the genetic association of APOE with delirium. For example, Adamis et al. and van Munster et al. reported an association between APOE and delirium [62-63], whereas studies by Bryson et al., Vasunilashorn et al., and another by Adamis et al. showed no relationship [64-66]. The current review was inconsistent with the results of Adamis et al. and van Munster but aligned with the findings of Bryson, Vasunilashorn et al., and Adamis et al.’s later study. Most publications investigated the serum levels of BDNF in various ways [67-71], and only a few studies focused on the genetic basis of BDNF in the context of delirium [72-73]. Therefore, this study recommends conducting more genetic association studies on BDNF candidate variants and individuals with DSD.

This review also introduced a miRNA (hsa-miR-17-5p) as a potential candidate circulating biomarker for detecting individuals with DSD. Evidence has demonstrated the roles of various miRNAs in dementia and delirium separately in both human and animal studies [74-75]. A recent report by Estfanous et al. presented an intriguing study on a mouse model and human tissues, showing elevated expression levels of MiR-17 in the microglia of Alzheimer’s patients, disrupting autophagy-mediated amyloid-beta degradation [76]. Very few studies exist regarding the miRNAs involved in delirium pathogenicity in circulating plasma. Wang et al. suggested that MiRNA-320 may contribute to the up-regulation of insulin-like growth factor-1 messenger RNA and APP protein levels, offering a new strategy for treating POD [77].

Despite the comprehensive search strategy and rigorous eligibility criteria, this review had some limitations. The quality of the included studies was not assessed; therefore, some studies may have a high risk of bias. Furthermore, the concept of personalized medicine is comparatively new, and there is limited consensus on its definition and implementation, which restricts the applicability of the study findings to clinical practice.

## Conclusions

Delirium is a brief mental state that can lead to hospitalization, functional disability, and death, and personalized medicine is accurate, cost-effective, and molecularly targeted. Delirium is more common in psychosis, anxiety, and psychoactive medications, and physical restraints can disrupt the sleep-wake cycle, causing melatonin disruption. Some 70% of delirious patients go undiagnosed, increasing hospitalization, functional disability, and death. Bioinformatics-based DSD studies revealed new gene-gene, protein-protein, gene-drug, and gene-microRNA interactions. Six essential genes, SNCA, APP, BDNF, DNMT1, APOE, and TNF, showed FDA-approved drugs with reliable pharmacological effects. Delirium management involves identifying and addressing risk factors, creating an optimal brain recovery environment, minimizing neurological consequences, managing patient concerns, monitoring for resolution, and educating caregivers about relapse. DSD biomarker studies from molecular bioinformatics are intriguing. We discovered that delirium-indicating serum biomarkers existed in three categories: those present or increased before disease initiation, those increasing with initiation and decreasing with recovery, and those related to disease outcomes and consequences. We found that infection/sIRS causes delirium. Genome-wide association studies discovered delirium risk loci. Plasma inflammation, brain injury, coagulation, and delirium duration are linked. In this article, we analyzed Alzheimer's and dementia POD biomarkers. The review proposed BDNF and APOE variants for personalized medicine-based DSD identification. Delirium and APOE genetics are inconsistent, but this review supported Bryson, Vasunilashorn, et al. Hsa-miR-17-5p is a DSD biomarker. Personalized medicine's definition and implementation limit the study's clinical relevance.

## References

[1] Soriano RP, Fernandez HM, Cassel CK, Leipzig RM (2007). Depression, dementia, and delirium. Fundamentals of Geriatric Medicine. A Case-Based Approach.

[2] Robinson TN, Eiseman B (2008). Postoperative delirium in the elderly: diagnosis and management. Clin Interv Aging.

[3] Nitchingham A, Caplan GA (2021). Current challenges in the recognition and management of delirium superimposed on dementia. Neuropsychiatr Dis Treat.

[4] Fortini A, Morettini A, Tavernese G, Facchini S, Tofani L, Pazzi M (2014). Delirium in elderly patients hospitalized in internal medicine wards. Intern Emerg Med.

[5] Fick DM, Hodo DM, Lawrence F, Inouye SK (2007). Recognizing delirium superimposed on dementia: assessing nurses' knowledge using case vignettes. J Gerontol Nurs.

[6] Uldall K, Williams BL, Dunn JD, Blackmore CC (2014). Association between combative behavior requiring intervention and delirium in hospitalized patients. J Hosp Med.

[7] Inouye SK, Director AB, Life HS (2014). The Short Confusion Assessment Method (Short CAM): Training Manual and Coding Guide. Boston, Hospital Elder Life Program.

[8] Lakhan P, Jones M, Wilson A, Courtney M, Hirdes J, Gray LC (2011). A prospective cohort study of geriatric syndromes among older medical patients admitted to acute care hospitals. J Am Geriatr Soc.

[9] Long SJ, Brown KF, Ames D, Vincent C (2013). What is known about adverse events in older medical hospital inpatients? A systematic review of the literature. Int J Qual Health Care.

[10] Iliffe S, Robinson L, Brayne C, Goodman C, Rait G, Manthorpe J, Ashley P (2009). Primary care and dementia: 1. diagnosis, screening and disclosure. Int J Geriatr Psychiatry.

[11] George J, Long S, Vincent C (2013). How can we keep patients with dementia safe in our acute hospitals? A review of challenges and solutions. J R Soc Med.

[12] Demeure MJ, Fain MJ (2006). The elderly surgical patient and postoperative delirium. J Am Coll Surg.

[13] Zhang JP, Lencz T, Geisler S, DeRosse P, Bromet EJ, Malhotra AK (2013). Genetic variation in BDNF is associated with antipsychotic treatment resistance in patients with schizophrenia. Schizophr Res.

[14] Cargnin S, Viana M, Sances G (2014). Combined effect of common gene variants on response to drug withdrawal therapy in medication overuse headache. Eur J Clin Pharmacol.

[15] Lane HY, Liu YC, Huang CL, Chang YC, Wu PL, Lu CT, Chang WH (2006). Risperidone-related weight gain: genetic and nongenetic predictors. J Clin Psychopharmacol.

[16] Domschke K, Lawford B, Laje G (2010). Brain-derived neurotrophic factor ( BDNF) gene: no major impact on antidepressant treatment response. Int J Neuropsychopharmacol.

[17] Wang XC, Xu DJ, Chen GH, Xia Q, Liu LN (2014). Association of 2 neurotrophic factor polymorphisms with efficacy of paroxetine in patients with major depressive disorder in a Chinese population. Ther Drug Monit.

[18] Kim BN, Cummins TD, Kim JW (2011). Val/Val genotype of brain-derived neurotrophic factor (BDNF) Val⁶⁶Met polymorphism is associated with a better response to OROS-MPH in Korean ADHD children. Int J Neuropsychopharmacol.

[19] Perroud N, Aitchison KJ, Uher R (2009). Genetic predictors of increase in suicidal ideation during antidepressant treatment in the GENDEP project. Neuropsychopharmacology.

[20] Yuan B, Sun X, Xu Z, Pu M, Yuan Y, Zhang Z (2020). Influence of genetic polymorphisms in homocysteine and lipid metabolism systems on antidepressant drug response. BMC Psychiatry.

[21] Geifman N, Brinton RD, Kennedy RE, Schneider LS, Butte AJ (2017). Evidence for benefit of statins to modify cognitive decline and risk in Alzheimer's disease. Alzheimers Res Ther.

[22] Braga IL, Silva PN, Furuya TK (2015). Effect of APOE and CHRNA7 genotypes on the cognitive response to cholinesterase inhibitor treatment at different stages of Alzheimer's disease. Am J Alzheimers Dis Other Demen.

[23] Loscalzo J, Fauci AS, Kasper DL, Hauser SL, Longo DL, Jameson JL (2022). Harrison's Principles of Internal Medicine. L., Longo, D. L., &amp; Jameson, J. L.

[24] Thom RP, Levy-Carrick NC, Bui M, Silbersweig D (2023). Delirium. Am J Psychiatry.

[25] Wan M, Chase JM. (2023). Delirium in older adults: Diagnosis, prevention, and treatment. BC Med J.

[26] Huang J (2023). Delirium - neurologic disorders. Merck Manuals Professional Edition.

[27] Lawlor PG, Bush SH (2014). Delirium diagnosis, screening and management. Curr Opin Support Palliat Care.

[28] Pan Y, Jiang Z, Yuan C (2018). Influence of physical restraint on delirium of adult patients in ICU: a nested case-control study. J Clin Nurs.

[29] Otahbachi M, Cevik C, Bagdure S, Nugent K (2010). Excited delirium, restraints, and unexpected death: a review of pathogenesis. Am J Forensic Med Pathol.

[30] Young J, Murthy L, Westby M, Akunne A, O'Mahony R (2010). Diagnosis, prevention, and management of delirium: summary of NICE guidance. BMJ.

[31] Devlin JW, Skrobik Y, Gélinas C (2018). Clinical practice guidelines for the prevention and management of pain, agitation/sedation, delirium, immobility, and sleep disruption in adult patients in the ICU. Crit Care Med.

[32] Downing LJ, Caprio TV, Lyness JM (2013). Geriatric psychiatry review: differential diagnosis and treatment of the 3 D's - delirium, dementia, and depression. Curr Psychiatry Rep.

[33] Inouye SK, Bogardus ST Jr, Charpentier PA (1999). A multicomponent intervention to prevent delirium in hospitalized older patients. N Engl J Med.

[34] Pisani MA, McNicoll L, Inouye SK (2003). Cognitive impairment in the intensive care unit. Clin Chest Med.

[35] Inouye SK (1998). Delirium in hospitalized older patients. Clin Geriatr Med.

[36] Morandi A, McCurley J, Vasilevskis EE (2012). Tools to detect delirium superimposed on dementia: a systematic review. J Am Geriatr Soc.

[37] Rincon HG, Granados M, Unutzer J (2001). Prevalence, detection and treatment of anxiety, depression, and delirium in the adult critical care unit. Psychosomatics.

[38] Figueroa-Ramos MI, Arroyo-Novoa CM, Lee KA, Padilla G, Puntillo KA (2009). Sleep and delirium in ICU patients: a review of mechanisms and manifestations. Intensive Care Med.

[39] Dessap AM, Roche-Campo F, Launay JM (2015). Delirium and circadian rhythm of melatonin during weaning from mechanical ventilation: an ancillary study of a weaning trial. Chest.

[40] Oh ES, Fong TG, Hshieh TT, Inouye SK (2017). Delirium in older persons: advances in diagnosis and treatment. JAMA.

[41] Kotfis K, van Diem-Zaal I, Williams Roberson S (2022). The future of intensive care: delirium should no longer be an issue. Crit Care.

[42] van der Kooi AW, Peelen LM, Raijmakers RJ (2015). Use of physical restraints in Dutch intensive care units: a prospective multicenter study. Am J Crit Care.

[43] Burry LD, Cheng W, Williamson DR (2021). Pharmacological and non-pharmacological interventions to prevent delirium in critically ill patients: a systematic review and network meta-analysis. Intensive Care Med.

[44] Neufeld KJ, Yue J, Robinson TN, Inouye SK, Needham DM (2016). Antipsychotic medication for prevention and treatment of delirium in hospitalized adults: a systematic review and meta-analysis. J Am Geriatr Soc.

[45] Al-Qadheeb NS, Skrobik Y, Schumaker G (2016). Preventing ICU subsyndromal delirium conversion to delirium with low-dose IV haloperidol: a double-blind, placebo-controlled pilot study. Crit Care Med.

[46] Shrestha P, Fick DM (2020). Family caregiver's experience of caring for an older adult with delirium: a systematic review. Int J Older People Nurs.

[47] Marcantonio ER, Rudolph JL, Culley D, Crosby G, Alsop D, Inouye SK (2006). Serum biomarkers for delirium. J Gerontol A Biol Sci Med Sci.

[48] de Jonghe A, Korevaar JC, van Munster BC, de Rooij SE (2010). Effectiveness of melatonin treatment on circadian rhythm disturbances in dementia. Are there implications for delirium? A systematic review. Int J Geriatr Psychiatry.

[49] van den Boogaard M, Kox M, Quinn KL (2011). Biomarkers associated with delirium in critically ill patients and their relation with long-term subjective cognitive dysfunction; indications for different pathways governing delirium in inflamed and noninflamed patients. Crit Care.

[50] Wijnia JW, Oudman E (2013). Biomarkers of delirium as a clue to diagnosis and pathogenesis of Wernicke-Korsakoff syndrome. Eur J Neurol.

[51] Hall RJ, Watne LO, Cunningham E (2018). CSF biomarkers in delirium: a systematic review. Int J Geriatr Psychiatry.

[52] Hov KR, Bolstad N, Idland AV (2017). Cerebrospinal fluid S100B and Alzheimer's disease biomarkers in hip fracture patients with delirium. Dement Geriatr Cogn Dis Extra.

[53] Henjum K, Quist-Paulsen E, Zetterberg H, Blennow K, Nilsson LN, Watne LO (2018). CSF sTREM2 in delirium-relation to Alzheimer's disease CSF biomarkers Aβ42, t-tau and p-tau. J Neuroinflamm.

[54] McCoy TH Jr, Hart K, Pellegrini A, Perlis RH (2018). Genome-wide association identifies a novel locus for delirium risk. Neurobiol Aging.

[55] Vasunilashorn SM, Ngo LH, Inouye SK (2020). Apolipoprotein E genotype and the association between C-reactive protein and postoperative delirium: Importance of gene-protein interactions. Alzheimers Dement.

[56] McNeil JB, Hughes CG, Girard T (2019). Plasma biomarkers of inflammation, coagulation, and brain injury as predictors of delirium duration in older hospitalized patients. PLoS One.

[57] Bowman K, Jones L, Pilling LC (2019). Vitamin D levels and risk of delirium: a mendelian randomization study in the UK Biobank. Neurology.

[58] Wang S, Lindroth H, Chan C (2021). A systematic review of delirium biomarkers and their alignment with the NIA-AA research framework. J Am Geriatr Soc.

[59] Wang P, Velagapudi R, Kong C (2020). Neurovascular and immune mechanisms that regulate postoperative delirium superimposed on dementia. Alzheimers Dement.

[60] Fong TG, Vasunilashorn SM, Gou Y (2021). Association of CSF Alzheimer's disease biomarkers with postoperative delirium in older adults. Alzheimers Dement (NY).

[61] Wang S, Greene R, Song Y (2022). Postoperative delirium and its relationship with biomarkers for dementia: a meta-analysis. Int Psychogeriatr.

[62] Adamis D, Treloar A, Martin FC, Gregson N, Hamilton G, Macdonald AJ (2007). APOE and cytokines as biological markers for recovery of prevalent delirium in elderly medical inpatients. Int J Geriatr Psychiatry.

[63] van Munster BC, Korevaar JC, Zwinderman AH, Leeflang MM, de Rooij SE (2009). The association between delirium and the apolipoprotein E epsilon 4 allele: new study results and a meta-analysis. Am J Geriatr Psychiatry.

[64] Bryson GL, Wyand A, Wozny D, Rees L, Taljaard M, Nathan H (2011). A prospective cohort study evaluating associations among delirium, postoperative cognitive dysfunction, and apolipoprotein E genotype following open aortic repair. Can J Anaesth.

[65] Vasunilashorn S, Ngo L, Kosar CM, Fong TG, Jones RN, Inouye SK, Marcantonio ER (2015). Does apolipoprotein E genotype increase risk of postoperative delirium?. Am J Geriatr Psychiatry.

[66] Adamis D, Meagher D, Williams J, Mulligan O, McCarthy G (2016). A systematic review and meta-analysis of the association between the apolipoprotein E genotype and delirium. Psychiatr Genet.

[67] Wyrobek J, LaFlam A, Max L (2017). Association of intraoperative changes in brain-derived neurotrophic factor and postoperative delirium in older adults. Br J Anaesth.

[68] Toft K, Tontsch J, Abdelhamid S, Steiner L, Siegemund M, Hollinger A (2019). Serum biomarkers of delirium in the elderly: a narrative review. Ann Intensive Care.

[69] Grandi C, Tomasi CD, Fernandes K (2011). Brain-derived neurotrophic factor and neuron-specific enolase, but not S100β, levels are associated to the occurrence of delirium in intensive care unit patients. J Crit Care.

[70] Williams J, Finn K, Melvin V, Meagher D, McCarthy G, Adamis D (2017). The association of serum levels of brain-derived neurotrophic factor with the occurrence of and recovery from delirium in older medical inpatients. Biomed Res Int.

[71] Li Y, Yu ZX, Ji MS (2019). A pilot study of the use of dexmedetomidine for the control of delirium by reducing the serum concentrations of brain-derived neurotrophic factor, neuron-specific enolase, and S100B in polytrauma patients. J Intensive Care Med.

[72] Matsushita S, Kimura M, Miyakawa T (2004). Association study of brain-derived neurotrophic factor gene polymorphism and alcoholism. Alcohol Clin Exp Res.

[73] Saito T, Braun PR, Daniel S (2020). The relationship between DNA methylation in neurotrophic genes and age as evidenced from three independent cohorts: differences by delirium status. Neurobiol Aging.

[74] Long JM, Maloney B, Rogers JT, Lahiri DK (2019). Novel upregulation of amyloid-β precursor protein (APP) by microRNA-346 via targeting of APP mRNA 5'-untranslated region: implications in Alzheimer's disease. Mol Psychiatry.

[75] Shan L, Ma D, Zhang C, Xiong W, Zhang Y (2017). miRNAs may regulate GABAergic transmission associated genes in aged rats with anesthetics-induced recognition and working memory dysfunction. Brain Res.

[76] Estfanous S, Daily KP, Eltobgy M (2021). Elevated expression of MiR-17 in microglia of Alzheimer's disease patients abrogates autophagy-mediated amyloid-β degradation. Front Immunol.

[77] Wang B, Yin Z, Lin Y (2022). Correlation between microRNA-320 and postoperative delirium in patients undergoing tibial fracture internal fixation surgery. BMC Anesthesiol.

